# Tumor Microenvironment-Associated Immune-Related Genes for the Prognosis of Malignant Pleural Mesothelioma

**DOI:** 10.3389/fonc.2020.544789

**Published:** 2020-09-16

**Authors:** Xiaoling Xu, Lei Cheng, Yun Fan, Weimin Mao

**Affiliations:** ^1^Department of Medical Oncology, The First Affiliated Hospital of Soochow University, Suzhou, China; ^2^Department of Medical Oncology, Institute of Cancer Research and Basic Medical Sciences of Chinese Academy of Sciences, Cancer Hospital of University of Chinese Academy of Sciences, Zhejiang Cancer Hospital, Hangzhou, China; ^3^Department of Thoracic Radiotherapy, Institute of Cancer Research and Basic Medical Sciences of Chinese Academy of Sciences, Cancer Hospital of University of Chinese Academy of Sciences, Zhejiang Cancer Hospital, Hangzhou, China; ^4^Department of Thoracic Surgery, Institute of Cancer Research and Basic Medical Sciences of Chinese Academy of Sciences, Cancer Hospital of University of Chinese Academy of Sciences, Zhejiang Cancer Hospital, Hangzhou, China

**Keywords:** malignant pleural mesothelioma, tumor microenvironment, ESTIMATE algorithm, immune signature, prognosis

## Abstract

Malignant pleural mesothelioma (MPM) is a rare but highly aggressive thoracic malignancy. ESTIMATE algorithm-derived immune scores are commonly used to quantify the immune and stromal components in tumors. Thus, this algorithm may help determine the tumor microenvironment (TME)-related gene expression profile associated with tumor immunity. This study aimed at mining public databases to determine a potential correlation between differentially expressed genes (DEGs) and survival in patients with MPM. We categorized patients from the Gene Expression Omnibus database according to their immune/stromal scores into high- and low-score groups. Functional enrichment analysis and the construction of protein–protein interaction networks showed that the DEGs identified were primarily involved in the TME. Furthermore, we validated these genes in an independent cohort of patients with MPM from The Cancer Genome Atlas database. DEG analysis showed that 29 DEGs were cancer driver genes. Subsequently, 14 TME-related genes, which have been previously neglected, were shown to exhibit significant prognostic potential in MPM. In conclusion, immune/stromal scores are novel predictors of a poor prognosis in patients with MPM. We identified DEGs that are involved in immunity against MPM and may contribute to patient survival. Owing to their potential as prognostic factors for MPM, these 14 TME-related genes need to be studied in detail in the future.

## Introduction

Malignant pleural mesothelioma (MPM) is a highly aggressive thoracic malignancy, with three major histological subtypes, namely, epithelioid, sarcomatoid, and biphasic mesotheliomas. MPM is a rare cancer, occurring in 30 out of a million individuals, although its incidence has slightly increased over the last decade (1). Chinese individuals are predominantly affected by MPM, owing to their exposure to high levels of asbestos (2, 3). Multimodality treatment (4), including surgical resection, radiation therapy, chemotherapy, and immunotherapy, is preferred for MPM because of the potential to increase efficacy and prognostic value. Thus, a multidisciplinary therapy-based model for the diagnosis and treatment of patients with MPM is extremely important (5–7).

Immunotherapy has become increasingly important in the management of cancers. Immune checkpoint inhibitors targeting programmed cell death protein-1 (PD-1), its ligand (PD-L1), and cytotoxic T-lymphocyte antigen 4 (CTLA-4) are commonly used as first-line therapeutics against various cancers (5, 8–10). Several clinical trials have evaluated the efficacy and safety of immunotherapy in patients with MPM (1, 3, 11, 12). Thus, the KEYNOTE-028 trial (1) showed potential clinical benefits of pembrolizumab, an anti-PD-L1 monoclonal antibody, in patients with MPM showing positive PD-L1 expression. Several ongoing phase II trials, including the KEYNOTE-158 basket trial (ClinicalTrials.gov Identifier: NCT02628067) and KEYNOTE-139 study (ClinicalTrials.gov Identifier: NCT02399371), are assessing the activity of pembrolizumab as a second-line therapy for MPM. The potential therapeutic benefit of combination immunotherapy (CTLA-4 and PD-L1 blockade) against MPM is under investigation, and thus far, the data from the CheckMate-743 (ClinicalTrials.gov Identifier: NCT02899299) and NIBIT-MESO-1 trials seem promising (3). There are only a few ongoing trials using a combination of immunotherapy and chemotherapy or surgery, or both, in patients with MPM. The DREAM trial is a multicenter, single-arm, open-label phase II study that aims to determine the effects of durvalumab in combination with chemotherapy for the treatment of MPM. Based on the data presented at the 2018 American Society of Clinical Oncology Conference, the therapeutic efficacy of this regimen seems promising.

The tumor immune microenvironment, including endothelial, stromal, and immune cells, plays a vital role in tumor surveillance and antitumor effects (2, 7). Understanding the correlation between tumor immunity and antitumor effectors in the MPM immune microenvironment is critical for enhancing the potential efficacy of immunotherapy (2, 7). Increasing evidence suggests that analysis of gene expression or copy numbers in cancer samples helps understand immune cell infiltration into the tumor microenvironment (TME). The ESTIMATE (Estimation of STromal and Immune cells in MAlignant Tumor tissues using Expression data) algorithm (5) can be used to analyze transcription profiles in tumor samples and their association with tumor cellularity and different infiltration characteristics. This algorithm generates scores to predict the levels of infiltrating stromal and immune cells and tumor heterogeneity (5).

In this study, we used ESTIMATE algorithm-derived immune scores of patients with MPM to generate a list of TME-associated genes based on the Gene Expression Omnibus (GEO) database. Subsequently, genes associated with poor outcomes were validated in a cohort of patients with MPM available from The Cancer Genome Atlas (TCGA) dataset.

## Materials and Methods

### Microarray Data Analysis

Gene expression profiles of 55 MPM tumor samples and paired normal tissues (for 41 tumor samples) were obtained from the GEO database under accession number GSE51024 (13). Gene expression was analyzed using the Affymetrix Human Genome U133 Plus 2.0 Array (Affymetrix, Inc., Santa Clara, CA, USA). The raw data were processed using the Robust Multi-Array Average method and the “Oligo” package from BioConductor (http://www.bioconductor.org) to normalize the data and annotate probe information.

### The Cancer Genome Atlas Data Analysis

Gene expression was analyzed using the Illumina HiSeq 2000 RNA sequencing platform at the University of North Carolina Genome Characterization Center. Level 3 data were obtained from the TCGA Data Coordinating Center. This dataset showed the predicted transcriptome profiles, presented as log_2_(x + 1)-transformed RSEM-normalized counts. Genes were mapped to human genome coordinates using HUGO probeMap. A method description is available from the University of North Carolina TCGA Genome Characterization Center. Demographic and clinical data, such as sex, age, histological type, survival, and outcome, were also obtained from TCGA.

### Initial Data Processing and Identification of Differentially Expressed Genes

Normalized signal intensities of the data were imported into BRBArrayTools (v.4.5; http://linus.nci.nih.gov/BRB-ArrayTools.html) for the initial processing and identification of differentially expressed genes (DEGs). A false discovery rate (FDR) of 0.05 was considered significant. DEGs were selected based on a fold change of ≥1.5 and FDR of < 0.05. Immune and stromal scores were calculated using the ESTIMATE algorithm (5). Heatmaps were generated, and clustering analyses were performed using the open-source web tool ClustVis (14).

### Enrichment Analysis of Differentially Expressed Genes

Functional enrichment analysis of DEGs was performed by DAVID (The Database for Annotation, Visualization and Integrated Discovery) to identify Gene Ontology (GO) categories by their biological processes (BPs), molecular functions (MFs), or cellular components (CCs). The DAVID database was also used to perform pathway enrichment analysis with reference from Kyoto Encyclopedia of Genes and Genomes (KEGG) pathways. FDR < 0.05 was used as the cut-off.

### Construction of a Protein–Protein Interaction Network

A total of 217,249 pairs of protein–protein interactions (PPIs) were downloaded from Reactome (v. 2014; http://www.reactome.org) (15). These pairwise correlations were derived from the PPI datasets from BioGrid (16), the Database of Interacting Proteins (17), Human Protein Reference Database (18), I2D (19), IntACT (20), and MINT (21), as well as from gene co-expression data derived from multiple high-throughput analyses, including yeast two-hybrid assays, mass spectrometry pull-down experiments, and DNA microarrays (22). The PPI network was constructed by importing the above interactions into Cytoscape (v. 3.2.1; http://www.cytoscape.org) (23).

### Identification of Network-Based Functional Modules

The Microarray Data Analysis tool from ReactomeFIViz was used for network-based functional analysis (24). After using DEGs as the input, we filtered the network modules using a cutoff of ≥2.

### Pathway and Gene Ontology Enrichment Analysis for Network-Based Functional Modules

ReactomeFIViz was used with Cytoscape for pathway and GO enrichment analysis (24). The sources of pathway annotations included CellMap (http://www.pathwaycommons.org/pc/dbSnapshot.do?snapshot_id = 8), Reactome (15), the KEGG (25), PANTHER (26), NCI–PID (27), and BioCarta (http://www.biocarta.com/genes/index.asp). An FDR of < 0.05 was used as the cut-off.

### Overall Survival Curve

Kaplan–Meier plots were generated to illustrate the relationship between patients' overall survival (OS) and gene expression levels of DEGs. The relationship was tested by log-rank test. *p* < 0.05 was used as the cut-off.

## Results

### Study Design and Workflow

**Figure 1:**
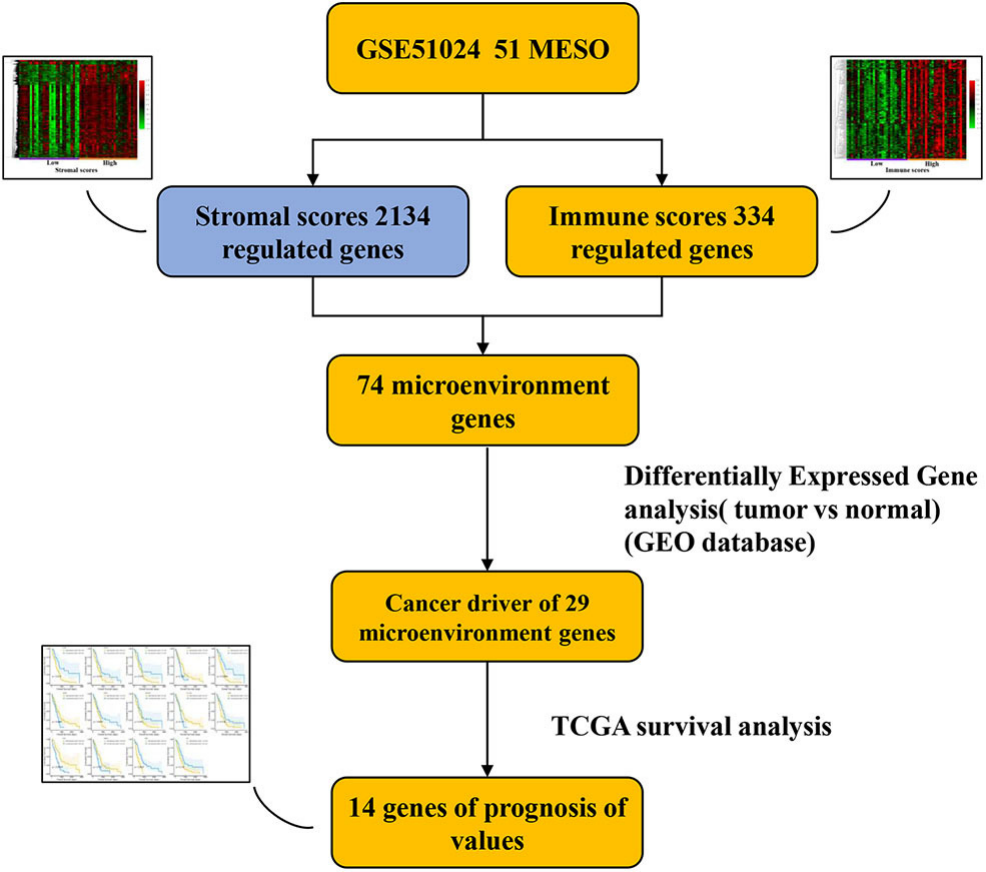
Workflow outline of the current study.

In this study, we identified, using a public database, TME-related genes that might affect the development and progression of MPM. Comparison of patients with high and low immune or stromal scores led to the identification of 74 stromal and immune cell-associated DEGs in the GEO database. GO and KEGG analyses showed that all 74 genes were associated with the TME. Analysis of the DEGs (tumor vs. normal) indicated that 29 DEGs were cancer driver genes, and survival analysis showed that 14 of these 29 genes were associated with a poor prognosis in patients with MPM. The prognostic values of the 14 TME-related genes were validated in patients with MPM from the TCGA database (Figure 1). Furthermore, a PPI network was constructed, in which seven network modules comprising 32 genes were obtained, to better understand the interactions among these DEGs.

### Correlation Between Differentially Expressed Genes and Immune and Stromal Scores Using the Gene Expression Omnibus Database

**Figure 2:**
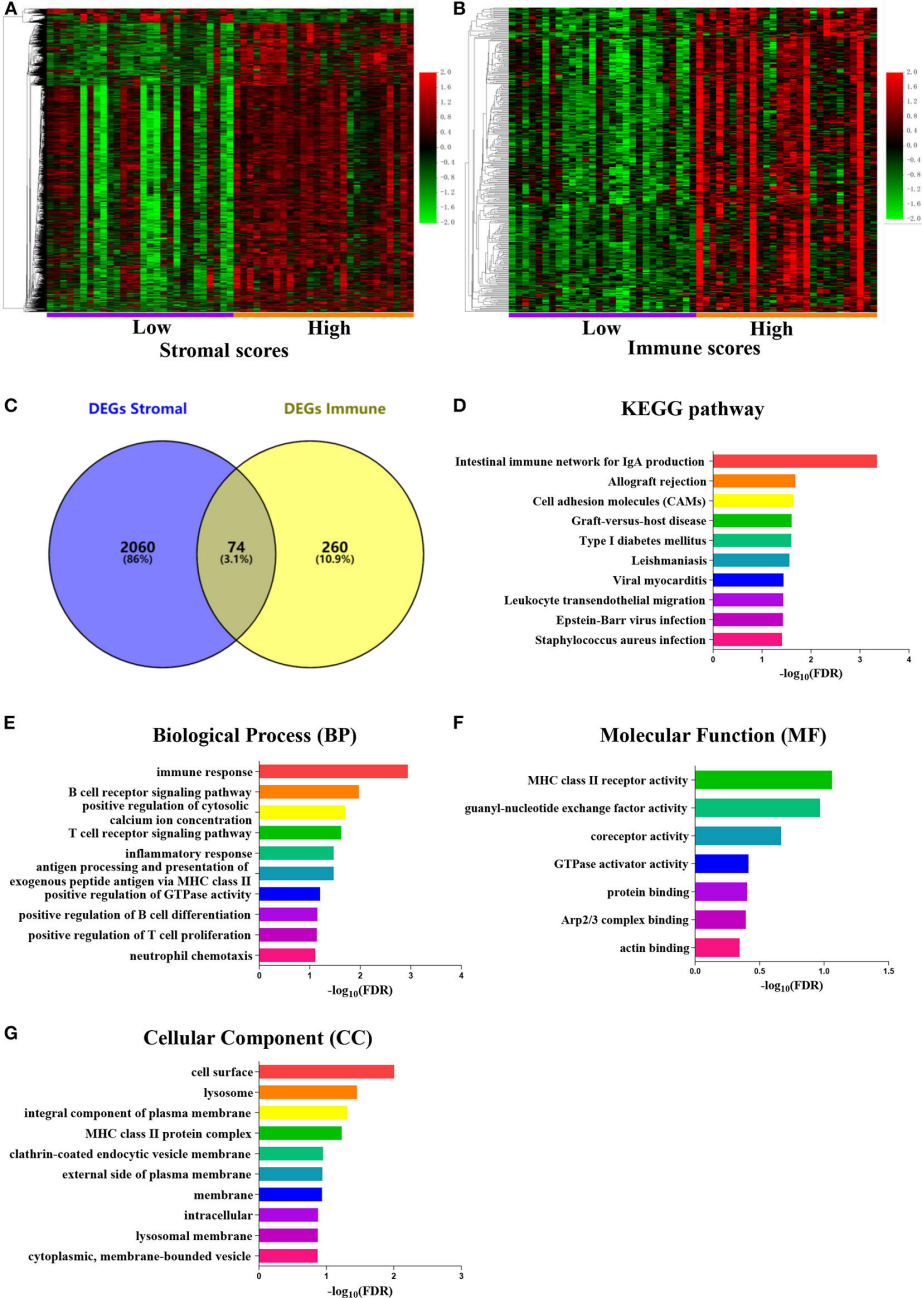
Comparison of the gene expression profile with immune scores and stromal scores in malignant pleural mesothelioma [Gene Expression Omnibus (GEO) database]. Heatmaps were drawn based on the average linkage method and Pearson distance measurement method. Genes with higher expression are shown in red, genes with lower expression are shown in green, and genes with mean expression level are shown in black. **(A)** Heatmap of the differentially expressed genes (DEGs) of stromal scores of the top half (high score) vs. bottom half (low score). False discovery rate (FDR) < 0.05, fold change ≥ 1.5. **(B)** Heatmap of the DEGs of immune scores of the top half (high score) vs. bottom half (low score). FDR < 0.05, fold change ≥ 1.5. **(C)** Venn diagrams showing the number of common DEGs in the stromal and immune score groups. **(D–G)** FDR of Kyoto Encyclopedia of Genes and Genomes (KEGG) and Gene Ontology (GO) enrichment analysis was acquired from DAVID functional annotation tool. *p* < 0.05.

To determine a correlation between DEG profiles and immune and/or stromal scores, we analyzed the data from a transcription microarray of 55 tumor tissues with paired normal tissues (for 41 tumor samples) from the GEO cohort. With the use of the BRBArrayTools software, 2,134 DEGs were identified based on the stromal scores, and 334 DEGs were identified based on the immune scores (Figures 2A–C). A total of 74 DEGs were validated using the Venn algorithm (Figure 2D).

### Functional Enrichment Analysis

Functional enrichment clustering analysis showed a significant number of genes at the intersection of the immune response. The KEGG pathway annotation analysis (Figure 2D) revealed genes associated with the intestinal immune network for IgA, cell adhesion molecules, and Epstein–Barr virus infection, among others. We selected the top 10 GO terms in the BP (Figure 2E), MF (Figure 2F), and CC (Figure 2G), among which immune response, B-cell receptor signaling pathway, T-cell receptor signaling pathway, and inflammatory response were the top GO terms identified.

### Significant Association of Immune and Stromal Scores With the Survival of Patients With Malignant Pleural Mesothelioma

The initial pathological diagnoses for 87 patients with MPM, for whom we obtained the gene expression profiles and clinical information from the TCGA database, were made between 1999 and 2013. Demographic and clinicopathological information, including sex, age, tumor location, history of asbestos exposure, histological classification, differentiation grade, pathological T, N, and M stages, and survival, was also retrieved from the database. However, we observed no correlations between the clinicopathological characteristics and immune and/or stromal scores (Supplementary Figure 1), except for the pathological T stage.

**Figure 3:**
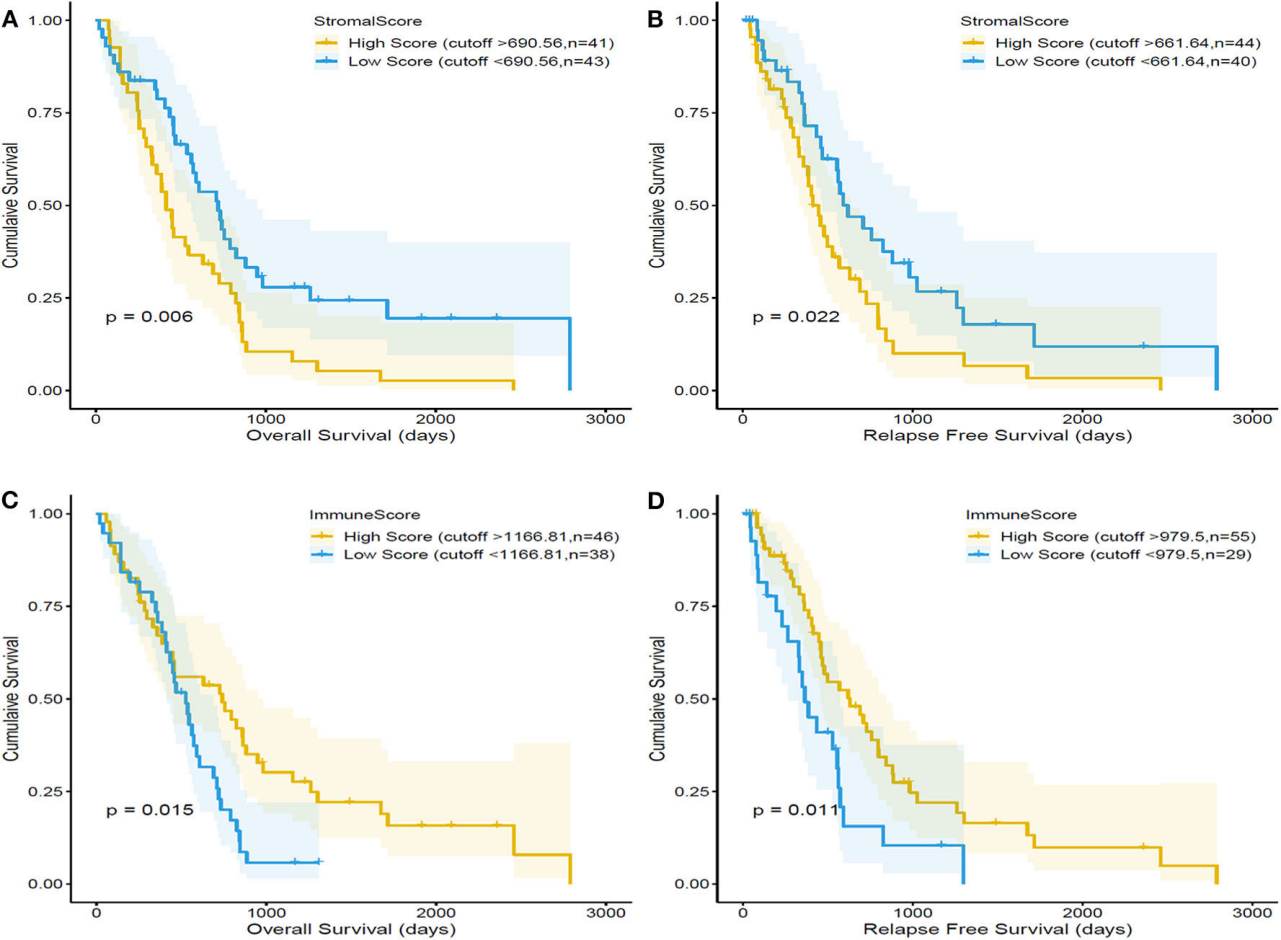
Correlation of stromal scores and immune scores with the overall survival and relapse-free survival in malignant pleural mesothelioma (MPM) using the dataset in The Cancer Genome Atlas (TCGA) database. MPM patients were divided into two groups based on their stromal scores. As shown in the Kaplan–Meier survival curve, the low stromal score group has significantly longer overall survival **(A)** and relapse-free survival than the high stromal score group **(B)**, as indicated by the log-rank test; *p*-value is 0.006 and 0.022, respectively. MPM patients were divided into two groups based on their immune scores. The high-immune-score group has significantly longer overall survival **(C)** and relapse-free survival **(D)** than the low-immune-score group; the log-rank test *p* = 0. 015 and *p* = 0.011.

Kaplan–Meier survival curves were used to evaluate the correlation between the immune and/or stromal scores and the prognosis of patients with MPM (Figure 3). The median OS of patients with low stromal scores (43 cases) was higher than that of patients with high stromal scores (43 cases) (719 vs. 414 days, respectively; *p* = 0.006, by a log-rank test). Similarly, the median relapse-free survival (RFS) of 40 cases with low stromal scores was higher than that of 44 cases with high stromal scores (620 vs. 414 days, respectively; *p* = 0.022, by a log-rank test). By contrast, the median OS of patients from the high-immune-score group (46 cases) was higher than that of patients from the low-immune-score group (38 cases) (620 vs. 434 days, respectively; *p* = 0.015, by a log-rank test). Meanwhile, the median RFS of patients from the high-immune-score group (55 cases) was higher than that of patients from the low-immune-score group (29 cases) (629 vs. 365 days, respectively; *p* = 0.011, by a log-rank test).

### Significant Association of Immune Function-Related Genes With the Survival of Patients With Malignant Pleural Mesothelioma

**Figure 4:**
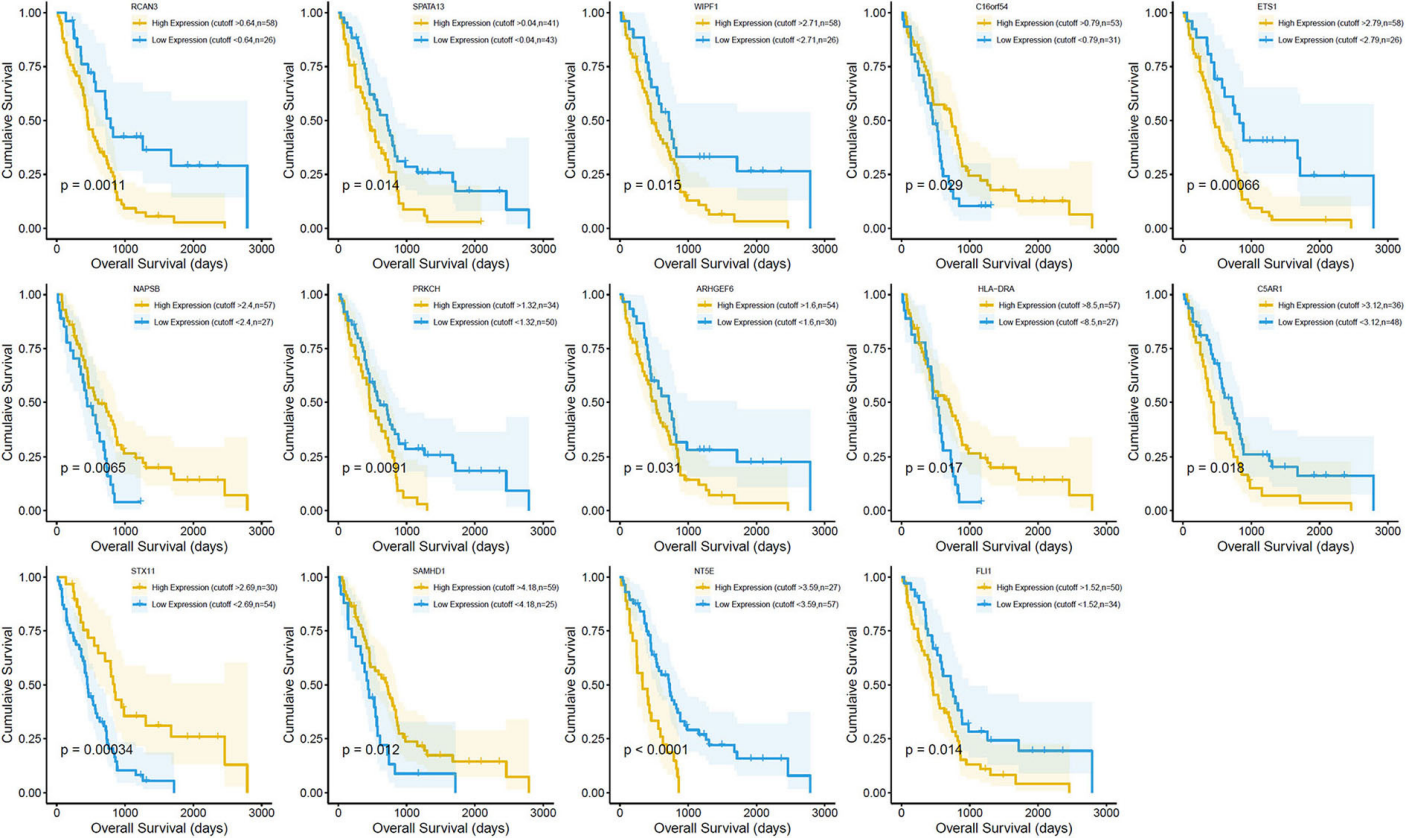
Correlation between the expression of the 14 individual differentially expressed genes (DEGs) with the overall survival [using The Cancer Genome Atlas (TCGA) dataset]. Kaplan–Meier survival curves were generated for the selected DEGs extracted from the comparison of high (yellow line) and low (blue line) gene expression groups. *p* < 0.05 in log-rank test. OS, overall survival in days.

**Figure 5:**
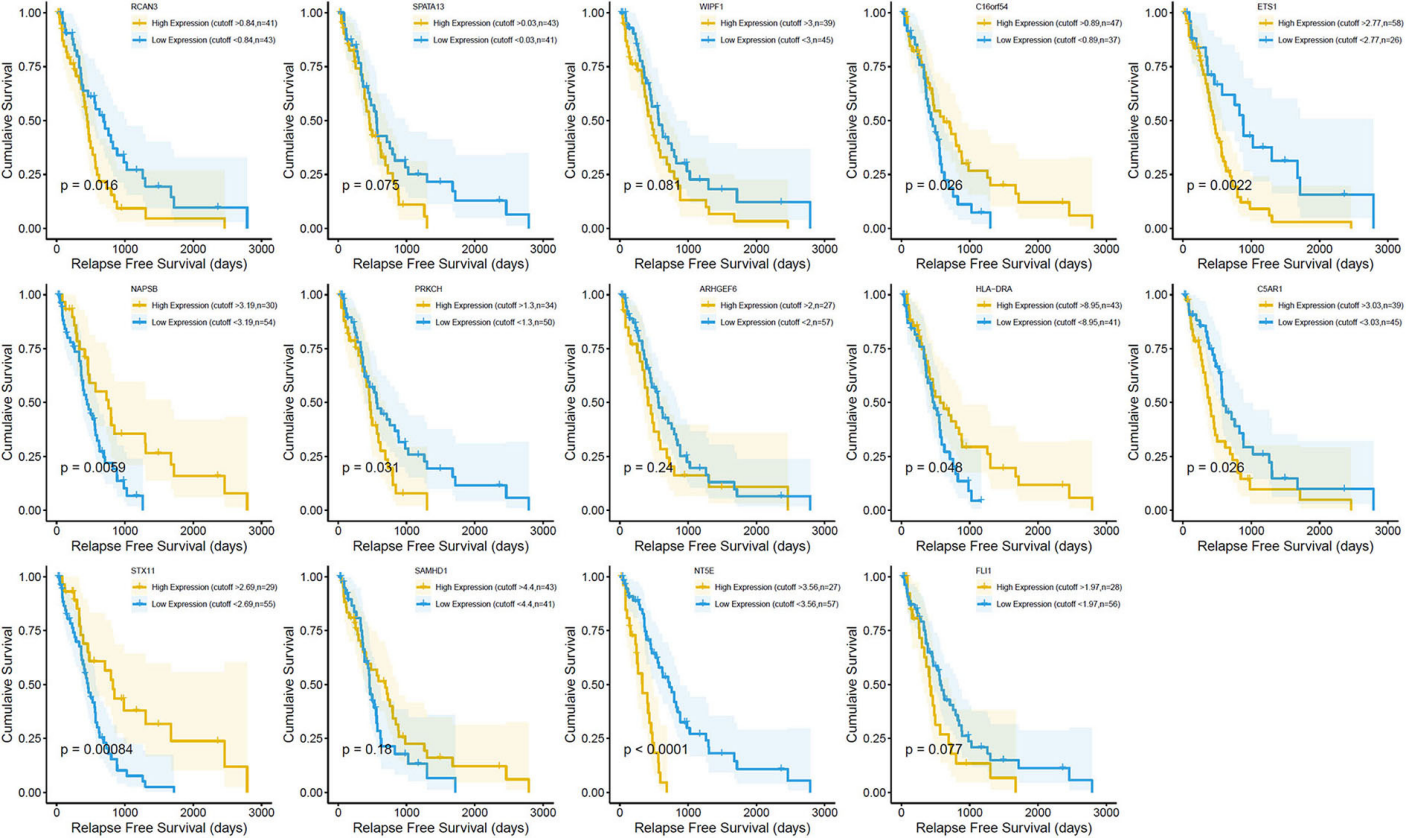
Correlation between the expression of 14 individual differentially expressed genes (DEGs) and the relapse-free survival (in days) in The Cancer Genome Atlas (TCGA) dataset. Kaplan–Meier survival curves were generated for the selected DEGs extracted from the comparison of high (yellow line) and low (blue line) gene expression groups. *p* < 0.05 in log-rank test. RFS, relapse-free survival in days.

**Figure 6:**
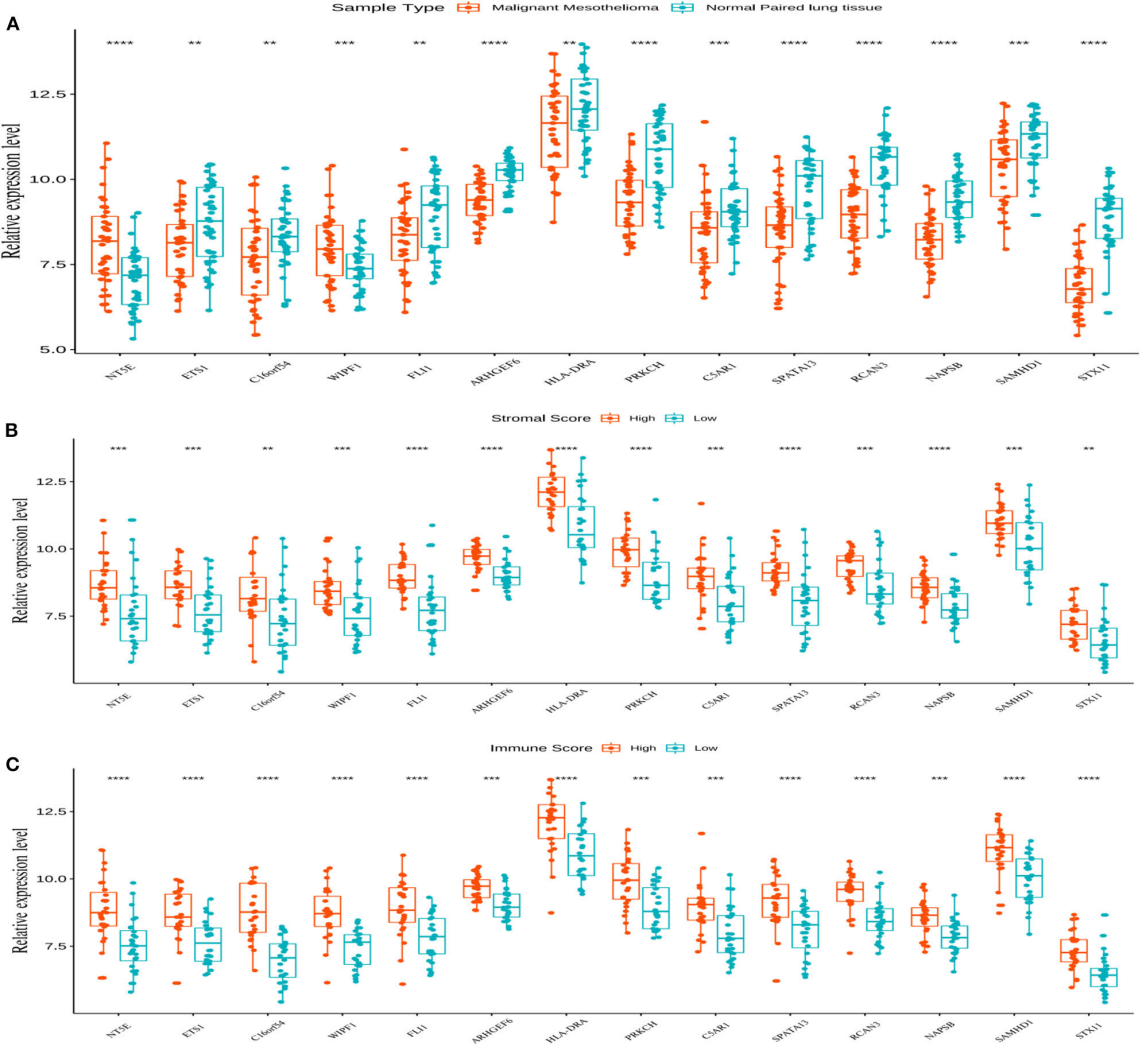
The expression of 14 genes and their relationship with the stromal and immune scores. **(A)** All 14 genes showed abnormal expression on comparing the malignant mesothelioma tissue with the normal paired lung tissue in the Gene Expression Omnibus (GEO) database. The box plot is used to show the relationship between the abnormal expression of the 14 genes and the stromal/immune scores **(B,C)**. **p < 0.01, ***p < 0.001, and ****p < 0.0001.

Survival analysis showed the presence of 29 cancer-specific DEGs. Of these, 14 immune-related genes were associated with a poor OS (Figure 4) and RFS (Figure 5), while 15 genes had no significant association with the survival of patients with MPM (Supplementary Figure 2). The 14 immune-related genes (Table 1) were *NT5E, ETS1, C16orf54, WIPF1, FLI1, ARHGEF6, HLA-DRA, PRKCH, C5AR1, SPATA13, RCAN3, NAPSB, SAMHD1*, and *STX11*; and their expression levels are shown in Figure 6A. The relationships between the immune/stromal scores and abnormal expression of these 14 immune-related genes are shown in Figures 6B,C.

**Table 1:** The information for the genes significant in MPM overall survival identified in TCGA.

**Gene**	**Official full name**	**Related pathway**	***p*-value**	**Method**
NT5E	5′-nucleotidase ecto	HIF-1-alpha transcription factor network	8.60E-06	Wilcoxon
**ETS1**	ETS proto-oncogene 1, transcription factor	AP-1 transcription factor network;C-MYB transcription factor network; BCR signaling pathway; HIF-1-alpha transcription factor network; HIF-2-alpha transcription factor network; IL4-mediated signaling events; Signaling events mediated by Hepatocyte Growth Factor Receptor (c-Met)	0.00249	Wilcoxon
C16orf54	Chromosome 16 open reading frame 54	None reported	0.00926	Wilcoxon
**WIPF1**	WAS/WASL interacting protein family member 1	Fc-epsilon receptor I signaling in mast cells	0.00079	Wilcoxon
**FLI1**	Fli-1 proto-oncogene, ETS transcription factor	DNA-binding transcription activator activity	0.00102	Wilcoxon
ARHGEF6	Rac/Cdc42 guanine nucleotide exchange factor 6	CDC42 signaling events	1.00E-10	Wilcoxon
**HLA-DRA**	Major histocompatibility complex, class II, DR alpha	IL12 signaling mediated by STAT4; IL12 signaling mediated by STAT4; CXCR4-mediated signaling events; TCR signaling in naïve CD4+ T cells; Cytokines and Inflammatory Response	0.00315	Wilcoxon
**PRKCH**	Protein kinase C eta	Calcium Regulation in the Cardiac Cell; G Protein Signaling Pathways; Wnt Signaling Pathway and Pluripotency	2.90E-09	Wilcoxon
C5AR1	Complement C5a receptor 1	Immune System; G alpha (i) signaling events; Class A/1 (Rhodopsin-like receptors)	0.00019	Wilcoxon
SPATA13	Spermatogenesis associated 13	Regulation of CDC42 activity; Regulation of RAC1 activity	4.80E-07	Wilcoxon
RCAN3	RCAN family member 3	None reported	6.90E-10	Wilcoxon
NAPSB	Napsin B aspartic peptidase, pseudogene	None reported	1.90E-07	Wilcoxon
SAMHD1	SAM and HD domain containing deoxynucleoside triphosphate triphosphohydrolase 1	Cytokine Signaling in Immune system; Immune System; Interferon Signaling; Interferon alpha/beta signaling; Metabolism	0.00014	Wilcoxon
STX11	Syntaxin 11	None reported	3.10E-08	Wilcoxon

*The p-value is for the differences in the expression of this gene in the GEO database. Genes in bold is also essential to in protein interaction analysis*.

### Protein–Protein Interaction and Hub Gene Identification

To explore the interplay among the 74 overlapping DEGs, a PPI network, which contained 32 nodes and 42 edges, was constructed using the Cytoscape software. In the PPI network, HLA-DRA, CD28, ITK, PTPRC, CXCR4, SYK, and PIK3CG were significant nodes because they had the largest numbers of connections with other nodes. Finally, the following nine TME-related hub genes were identified: *HLA-DRA, SYK, CXCR4, ITK, PTPRC, HLA-DPB1, PIK3CG, ETS1*, and *HCLS1*.

**Figure 7:**
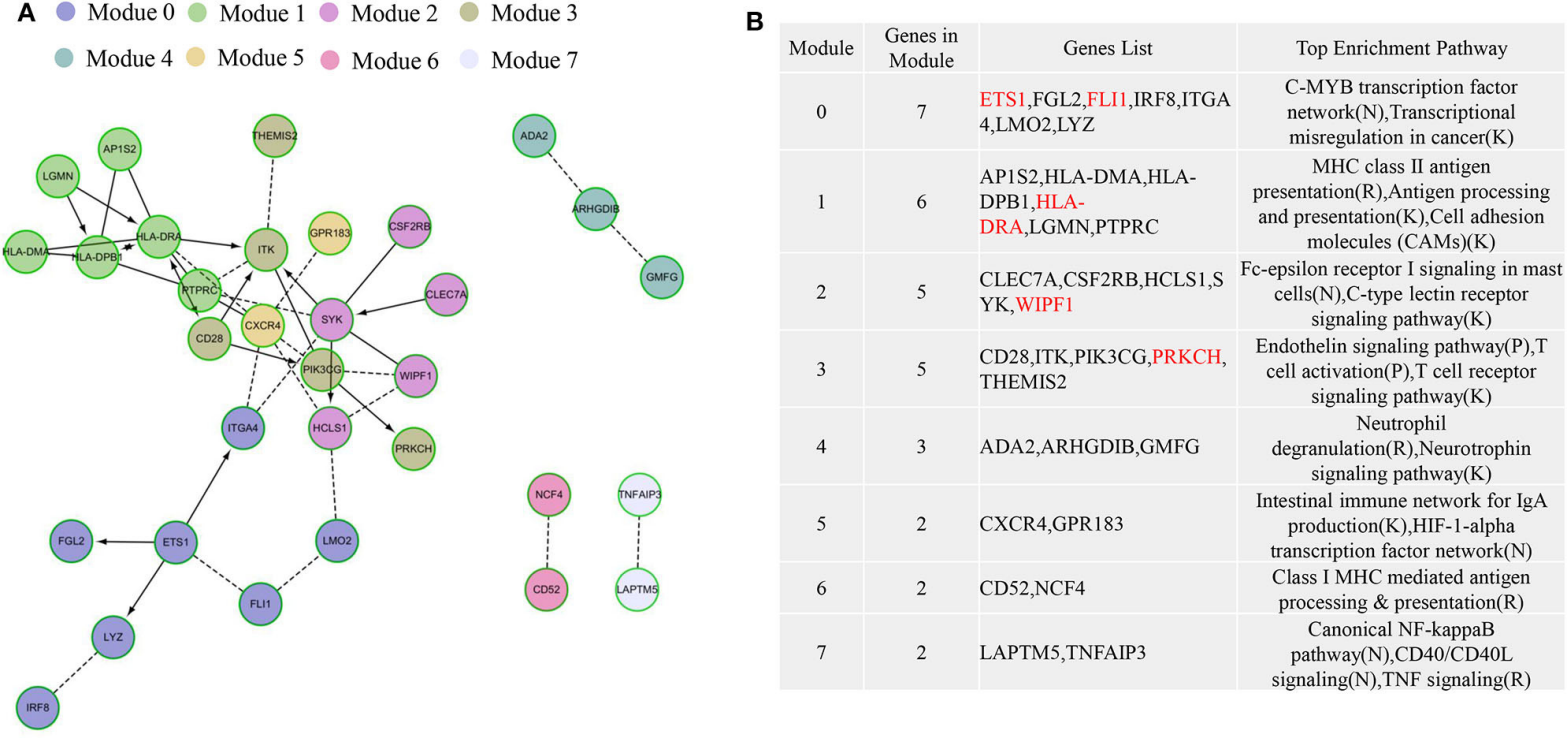
The protein–protein interaction (PPI) networks for the 74 differentially expressed genes (DEGs) in malignant pleural mesothelioma (MPM) patients. The functional interaction (FI) network constructed using immune-related genes in MPM. **(A)** The generated seven network modules comprise 32 genes, which are shown in different colors in different network modules. **(B)** A functional enrichment analysis of these modules based on pathway annotation. Genes in red are significant in survival analysis.

Functional enrichment analysis using ReactomeFIViz showed that the genes were involved in the C-MYB transcription factor network, MHC class II antigen presentation (R), antigen processing and presentation (K), cell adhesion molecule (CAM) interactions, Fc-epsilon receptor I signaling in mast cells (N), C-type lectin receptor signaling pathway (K), endothelin signaling pathway (P), T-cell activation (P), and T-cell receptor signaling pathway (K), most of which are related to immunity (Figure 7).

## Discussion

It has been previously shown that molecular features of tumors can affect immune responses and the TME (5, 28). Therefore, identifying TME-related genes that determine the prognosis of MPM is crucial to preventing cancer progression and managing the potential immune response. In this study, we used the ESTIMATE algorithm (5) to screen for tumor immune-related genes in the GEO database and validate the prognostic values of genes in the TCGA database. To the best of our knowledge, this is the first report to demonstrate the involvement of 14 immune-related genes in MPM.

The composition of the TME may influence cancer development (29). Interactions between cancer stem cells and immune cells are important in the carcinogenesis of MPM (30). The TME may also be associated with the prognosis of MPM and may determine the potential efficacy of anticancer therapies (31). Immunotherapy exploits these mechanisms and imparts antitumor effects (32). There are multiple reports on the development of novel therapeutic strategies based on targeting the TME (29, 33). Recently, encouraging results of a first-line dual-immune phase III clinical trial on MPM were released at the 2020 World Conference on Lung Cancer, which are expected to break the 15-year deadlock without new drugs. The CheckMate-743 phase III clinical study showed that nivolumab injection, combined with ipilimumab, could significantly improve the OS of patients with previously untreated, unresectable MPM. At the shortest follow-up of 22 months, nivolumab, combined with ipilimumab, reduced the patient's risk of death by 26%. The patient's median OS was 18.1 months, compared with 14.1 months in the chemotherapy group (hazard ratio: 0.74; 96.6% confidence interval: 0.60–0.91; *p* = 0.002). The 2-year survival rate of patients in the nivolumab plus ipilimumab group was 41%, compared with 27% in the chemotherapy group (34). This study suggested that checkpoint inhibitors might be a potential effective treatment for MPM. However, more clinical trials, with OS as a primary end point, investigate the efficacy of immunotherapy compare with the best treatment (pemetrexed + cisplatin ± bevacizumab) for MPM is needed.

In our study, we aimed to explore whether genes related to the tumor immune microenvironment might predict the prognosis of patients with MPM. These genes might help to select the potential beneficiaries of immunotherapy. Five of the 14 immune-related genes (*ETS1, FLI1, WIPF1, HLA-DRA*, and *PRKCH*) represented essential elements in the PPI network. ETS1 (also known as p54 and EWSR2) is a member of the ETS family of transcription factors, which are involved in stem cell development, cell senescence and death, and tumorigenesis. ETS1 functions as an oncogene and is a crucial regulator of phenomena involved in tumorigenesis, such as the mesenchymal phenotype in various tumors, including head and neck squamous cell carcinoma (35), breast cancer (36), prostate cancer (37), and glioblastoma (38). ETS1 also functions in several immune-related pathways, including the C-MYB transcription factor network, BCR signaling, HIF-1α transcription factor network, HIF-2α transcription factor network, and IL4-mediated signaling (https://pubchem.ncbi.nlm.nih.gov/source/Pathway%20Interaction%20Database). FLI1 (also known as SIC-1 and BDPLT21) is a transcription factor containing an ETS DNA-binding domain; it is involved in gene fusions (39). FLI1 is associated with a poor prognosis in multiple tumors, including non-small-cell lung cancer (40), breast cancer (41), and acute myeloid leukemia (42). WIPF1 (also known as PRPL-2, WAS2, WASPIP, and WIP) is vital to the organization of the actin cytoskeleton. WIPF1 is involved in Fc-epsilon receptor I signaling, which is an important immune-related pathway, in mast cells. HLA-DRA, an HLA class II alpha-chain paralog, is involved in several immune-related pathways, such as STAT4-mediated IL12 signaling, CXCR4-mediated signaling, TCR signaling in naïve CD4^+^ T cells, and cytokine inflammatory responses. PRKCH is a protein kinase C and a member of the PKC family of proteins. PRKCH phosphorylates a wide variety of targets and is involved in diverse signaling pathways, including calcium regulation in cardiac cells, G-protein signaling pathways, the Wnt signaling pathway, and pluripotency. Taken together, these genes show prognostic potential and deserve further exploration to determine their potential as therapeutic targets in MPM.

The significance of this study is that we identified 14 TME-related genes. These genes are involved in immune responses, may predict the survival of patients with MPM, and may also play a role as biomarkers of the sensitivity to immunotherapy. Moreover, we validated these results in two databases. However, because of the low incidence of mesothelioma, it is very difficult to collect fresh clinical samples to verify our results.

Taken together, these identified genes show promise as prognostic markers and immune effectors. To the best of our knowledge, this is the first study to report that immune and stromal scores can predict the prognosis of MPM. High-immune and low-stromal scores are predictive of a good prognosis of MPM in patients. However, most clinicopathological characteristics, including the history of asbestos exposure, histological classification, differentiation grade, and pathological N stage, have no impact on the prognosis of MPM.

## Conclusion

In summary, our findings indicate the importance of TME-related genes that are involved in immune-related pathways in the prognosis of MPM and serve as potential predictors to improve the efficacy of precision immunotherapy. Based on the ESTIMATE algorithm, immune and stromal scores were calculated and found to be useful in determining the prognosis of patients with MPM. The DEGs were validated in two independent cohorts, from the GEO and TCGA databases. As the involvement of these genes in MPM was not confirmed in clinical samples from China, further studies are warranted in the future to confirm their roles in MPM.

## Data Availability Statement

All datasets generated for this study are included in the article/Supplementary Material.

## Author Contributions

XX and WM guaranteed the integrity of the manuscript and were involved in the conceptualization and design of the study. YF and LC contributed to the data collection, analysis, and interpretation. All the authors contributed to writing and revising the manuscript. The final manuscript has been read and approved by all authors.

## Conflict of Interest

The authors declare that the research was conducted in the absence of any commercial or financial relationships that could be construed as a potential conflict of interest.

## Supplementary Material

Supplementary Figure 1 The relationship between the clinicopathological characters and immune and/or stromal scores. Box plot showed that there is no relationship between the clinicopathological characters and the immune and/or stromal scores except pathologic T. Only pathologic T has a significant correlation with stromal scores. Ns means not significant, *means significant.

Supplementary Figure 2 Correlation between the expression of the 13 individual DEGs in the relapse free survival and overall survival (in days) in TCGA dataset. Kaplan-Meier survival curves were generated for the selected DEGs from the comparison of high (yellow line) and low (blue line) gene expression groups. All the 13 individual DEGs had no impact on the clinical outcome of malignant pleural mesothelioma patients. *P* < 0.05 in Log-rank test. RFS, relapse free survival in days; OS, overall survival. Note: Two genes lake of survival data in TCGA database.
