# Supplementary figures and images for: Tumor Microenvironment-Associated Immune-Related Genes for the Prognosis of Malignant Pleural Mesothelioma
Source: Front Oncol. 2020 Sep 16;10:544789. doi: 10.3389/fonc.2020.544789 (PMC7526499; doi:10.3389/fonc.2020.544789)

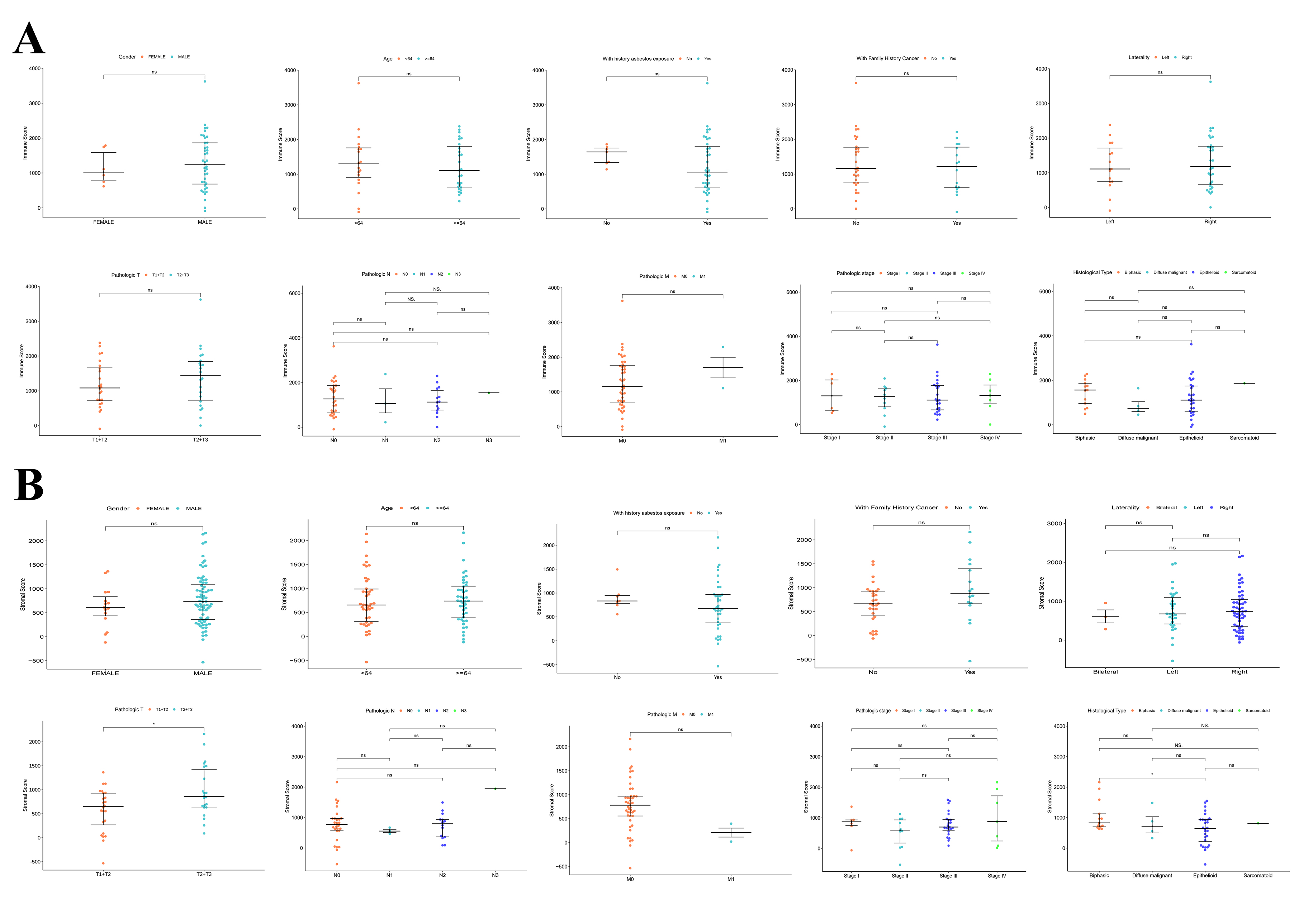

Supplement: Supplementary Figure 1 — The relationship between the clinicopathological characters and immune and/or stromal scores. Box plot showed that there is no relationship between the clinicopathological characters and the immune and/or stromal scores except pathologic T. Only pathologic T has a significant correlation with stromal scores. Ns means not significant, *means significant. [file Image_1.jpeg]

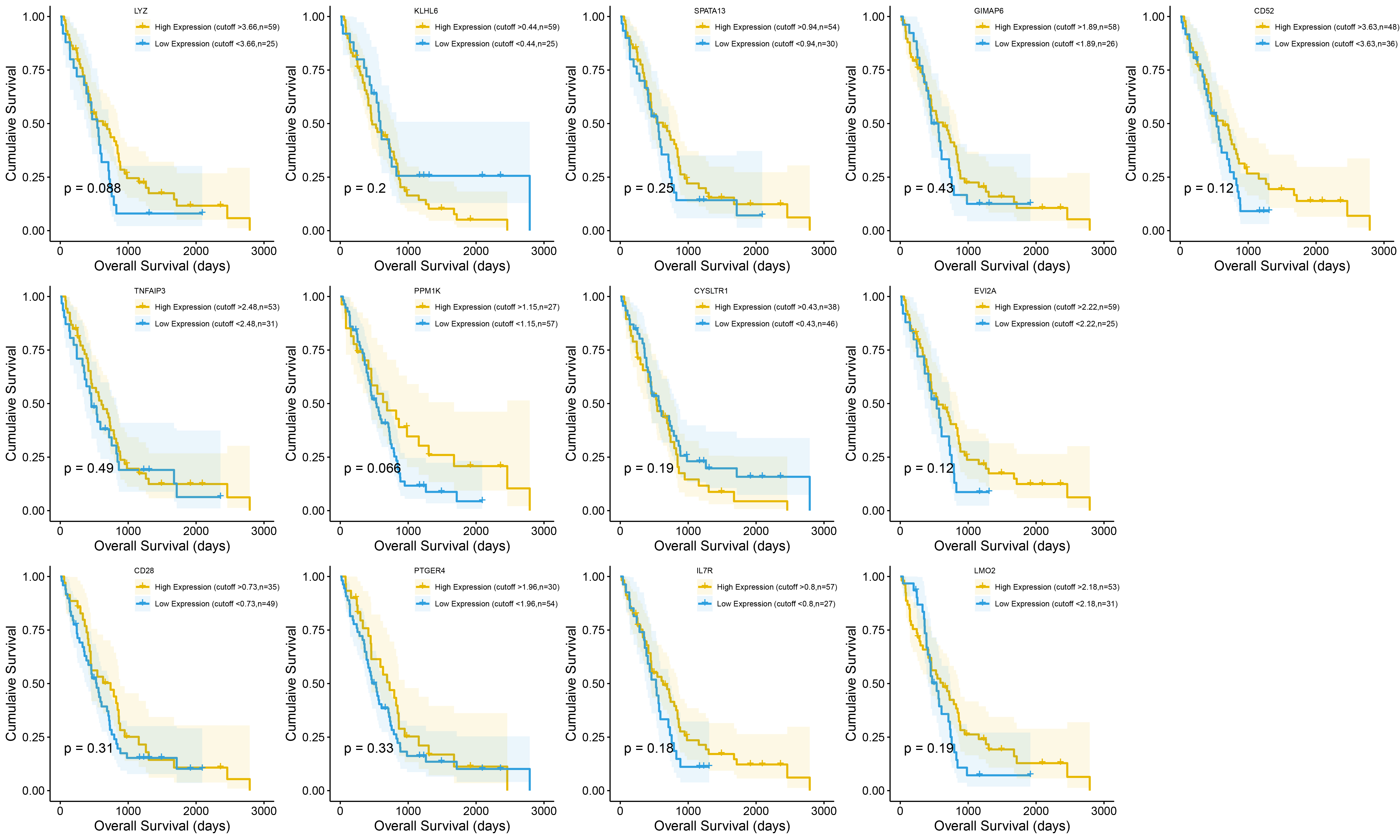

Supplement: Supplementary Figure 2 — Correlation between the expression of the 13 individual DEGs in the relapse free survival and overall survival (in days) in TCGA dataset. Kaplan-Meier survival curves were generated for the selected DEGs from the comparison of high (yellow line) and low (blue line) gene expression groups. All the 13 individual DEGs had no impact on the clinical outcome of malignant pleural mesothelioma patients. P < 0.05 in Log-rank test. RFS, relapse free survival in days; OS, overall survival. Note: Two genes lake of survival data in TCGA database. [file Image_2.jpeg]
